# A Pedometer-Guided Physical Activity Intervention for Obese Pregnant Women (the Fit MUM Study): Randomized Feasibility Study

**DOI:** 10.2196/15112

**Published:** 2020-05-26

**Authors:** Jai N Darvall, Andrew Wang, Mohamed Nusry Nazeem, Cheryce L Harrison, Lauren Clarke, Chennelle Mendoza, Anna Parker, Benjamin Harrap, Glyn Teale, David Story, Elizabeth Hessian

**Affiliations:** 1 Department of Anaesthesia and Pain Management Royal Melbourne Hospital Melbourne Australia; 2 Centre for Integrated Critical Care University of Melbourne Melbourne Australia; 3 Melbourne Medical School University of Melbourne Melbourne Australia; 4 Monash Centre for Health Research and Implementation School of Public Health and Preventive Medicine Monash University Melbourne Australia; 5 Department of Physiotherapy Western Health Melbourne Australia; 6 Melbourne Epicentre University of Melbourne Melbourne Australia; 7 Department of Women’s and Children’s Services, Western Health Melbourne Australia; 8 Department of Anaesthesia and Pain Management, Western Health Melbourne Australia

**Keywords:** gestational weight gain, pregnancy, maternal obesity, lifestyle intervention, pedometer

## Abstract

**Background:**

Obesity in pregnancy is a growing problem worldwide, with excessive gestational weight gain (GWG) occurring in the majority of pregnancies. This significantly increases risks to both mother and child. A major contributor to both prepregnancy obesity and excessive GWG is physical inactivity; however, past interventions targeting maternal weight gain and activity levels during the antenatal period have been ineffective in women who are already overweight. Pedometer-guided activity may offer a novel solution for increasing activity levels in this population.

**Objective:**

This initial feasibility randomized controlled trial aimed to test a pedometer-based intervention to increase activity and reduce excessive GWG in pregnant women.

**Methods:**

We supplied 30 pregnant women with obesity a Fitbit Zip pedometer and randomized them into 1 of 3 groups: control (pedometer only), app (pedometer synced to patients’ personal smartphone, with self-monitoring of activity), or app-coach (addition of a health coach–delivered behavioral change program). Feasibility outcomes included participant compliance with wearing pedometers (days with missing pedometer data), data syncing, and data integrity. Activity outcomes (step counts and active minutes) were analyzed using linear mixed models and generalized estimating equations.

**Results:**

A total of 30 participants were recruited within a 10-week period, with a dropout rate of 10% (3/30; 2 withdrawals and 1 stillbirth); 27 participants thus completed the study. Mean BMI in all groups was ≥35 kg/m^2^. Mean (SD) percentage of missing data days were 23.4% (20.6%), 39.5% (32.4%), and 21.1% (16.0%) in control, app group, and app-coach group patients, respectively. Estimated mean baseline activity levels were 14.5 active min/day and 5455 steps/day, with no significant differences found in activity levels between groups, with mean daily step counts in all groups remaining in the sedentary (5000 steps/day) or low activity (5000-7499 steps/day) categories for the entire study duration. There was a mean decrease of 7.8 steps/day for each increase in gestation day over the study period (95% CI 2.91 to 12.69, *P*=.002).

**Conclusions:**

Activity data syncing with a personal smartphone is feasible in a cohort of pregnant women with obesity. However, our results do not support a future definitive study in its present form. Recruitment and retention rates were adequate, as was activity data syncing to participants’ smartphones. A follow-up interventional trial seeking to reduce GWG and improve activity in this population must focus on improving compliance with activity data recording and behavioral interventions delivered.

**Trial Registration:**

Australian and New Zealand Clinical Trials Registry ACTRN12617000038392; https://www.anzctr.org.au/Trial/Registration/TrialReview.aspx?id=370884

## Introduction

Obesity in pregnancy is an endemic and growing problem worldwide. In Australia, 50% of all women who become pregnant are either overweight (BMI 25-30 kg/m^2^) or women with obesity (BMI≥30 kg/m^2^), corresponding with other developed countries [1-3]. Excess gestational weight gain (GWG) above US Institute of Medicine recommendations (which for women with obesity should not exceed 9 kg) occurs in the majority of pregnancies, with every kilogram above recommendations increasing adverse outcomes by 10% [4-6]. Excessive GWG in the presence of preexisting obesity exacerbates health risks for mother and child, including increased rates of gestational hypertension and diabetes, cesarean delivery, perinatal mortality, and neonatal hypoglycemia, jaundice, and admission to neonatal intensive care [1].

Although a complex problem, a significant contributor to prepregnancy obesity and excessive GWG is physical inactivity; similarly, interventions that have succeeded in increasing physical activity through exercise have been associated with a commensurate reduction in GWG in pregnant women. A recent Cochrane review encompassing over 14,000 women across 49 randomized controlled trials found a pooled reduction of 21% in excessive GWG with exercise interventions [7-9]. The evidence for increasing normal physical activity during the day, however, is less robust. World Health Organization recommendations for adults include at least 150 min of moderate-intensity, or at least 75 min of vigorous-intensity, aerobic physical activity per week, or a combination of both [10]. During pregnancy, targets for moderate-intensity physical activity are the same in the absence of contraindications [11,12]. Evidence also suggests pregnant women with obesity are less active than their pregnant normal weight counterparts; 2 recent studies using pedometers to examine activity levels in overweight and pregnant women with obesity, for example, demonstrated mean activity levels in the *sedentary* range (5000 steps/day) [13-16].

How to address this increasing problem is not clear. Frustratingly, despite the benefit of exercise in reducing GWG presented above, interventions seeking to target an increase in activity levels during the antenatal period have commonly failed in reducing GWG in the cohort of women who are already overweight [7,17]. However, there is emerging evidence that pedometer-guided activity interventions may be successful in increasing activity levels in both pregnant and nonpregnant populations [18]. The latest generation of pedometers also have the capacity to automatically upload activity information via smartphone to enable data capture and daily monitoring, which can be used to provide remote feedback to patients. Increasing smartphone ownership worldwide means there is scope for broad population reach, potentially overcoming some of the barriers to engagement with traditional models of health care, such as transport, cost, and rigidity of appointment times. What is not well understood from previous research, however, is the efficacy of smartphone app, data capturing availability, and biofeedback provided in the context of interventions to optimize GWG.

Having previously demonstrated the acceptability and utility of the Fitbit Zip pedometer as a remote activity monitoring device in a pilot study [19], we conducted a feasibility randomized controlled trial of a pedometer-based intervention in a cohort of pregnant women with obesity. In particular, we aimed to evaluate the feasibility of self-monitoring of activity levels via the Fitbit Zip pedometer and the additional role of a behavioral intervention in reducing the incidence of excessive GWG.

## Methods

This randomized, controlled feasibility trial was conducted in the Department of Anesthesia and Pain Management, Sunshine Hospital, Victoria, Australia. Approval was gained from the hospital Human Research and Ethics Committee (December 22, 2016, Human Research and Ethics Committee approval number HREC/16/MH/320), and the trial was registered with the Australian and New Zealand Clinical Trials Registry (ACTRN12617000038392, January 10, 2017). Female patients aged ≥18 years with BMI (weight in kg/height in m^2^) ≥30 kg/m^2^ with the availability of a smartphone capable of allowing Fitbit data uploading (eg, Apple iPhone or Android operating system equipped phone) were eligible for enrollment between gestational week 12 and 16. A convenience sample of 30 nonconsecutive patients attending the antenatal clinic was enrolled between March and May 2017 based on the availability of study investigators, after written informed consent. Patients were excluded if they had preeclampsia, twin or multiple pregnancies, preterm rupture of membranes, incompetent cervix/cerclage, or if they had a joint or muscle disorder sufficient to impair walking to a target of 10,000 steps daily. Patients were randomized to 1 of 3 groups (2 interventional and 1 control) via a computerized random number generator, with sequentially numbered envelopes used for allocation concealment. Because of the nature of the intervention, neither patients nor study investigators were blind to group allocation.

### Intervention

All patients were supplied with a Fitbit Zip pedometer and instructed to wear it daily on the waistband of clothing during waking hours. The pedometer measured step counts and minutes each day spent at various activity levels, characterized as either *sedentary*, *lightly*, *fairly*, or *very* active based on the cadence of steps recorded. *Active* minutes were commenced once activity exceeded 3 metabolic equivalents for ≥10 min [20]. Patients in the app and app-coach groups also had the pedometer synced to their personal smartphone via the Fitbit app (Fitbit Inc, San Francisco, California, USA), allowing automatic daily uploading of activity data. Each patient was registered on this platform under a de-identified email address. We considered days with >1000 steps reported as indicative of a day wearing the pedometer. Days with <1000 steps reported were censored as missing. Group conduct was as follows: (1) in the *control group*, the pedometer display was obscured using tamperproof tape, blinding patients to daily steps, and active minutes. To further ensure blinding, pedometers in the control group were not linked to participants’ smartphones, but instead were synced manually by study investigators at clinic appointments; (2) in the *app-only intervention group (app group)*, the pedometer was synced to patients’ personal smartphones, with patients encouraged to self-monitor daily step counts and activity minutes via the pedometer display or the Fitbit app; and (3) in the *app and coach intervention group (app-coach group)*, in addition to the intervention in the app group above, patients were administered a behavioral change program delivered by trained health coaches. This consisted of an initial 1-hour face-to-face session between 16 and 20 weeks of gestation at which goal setting for activity in pregnancy was discussed, including challenges and barriers to achievement, and *specific*, *measurable*, *achievable*, *relevant*, and *time-bound* [21] objectives were set. Patients then had 3 follow-up health coach 20-min telephone sessions at 24, 28, and 32 weeks of gestation, during which pedometer activity levels were reviewed, and strategies to achieve targets reinforced. This included exploring and resolving ambivalence, providing encouragement, and ensuring skills were practiced and action plans completed. The intervention was based on self-determination theory, including action planning, goal setting, and self-monitoring, all factors in long-term behavioral change [22]. The aims were to educate women about the importance of physical activity and healthy eating during pregnancy (commensurate with the guidelines below), the balance between energy intake and expenditure, and removing misconceptions and increasing confidence about engaging in physical activity throughout pregnancy (including advice on activity targets below and reassurance about the safety of vigorous exercise). In the event of patients not being contactable for follow-up telephone sessions, a total of 3 attempted phone calls were made over 2 weeks. The health coaches (CM and LC) were trained over 2 weeks in the delivery of the intervention, including motivational interviewing, by the developer of the program (CLH).

All patients, regardless of group allocation, were provided with written resources at enrollment, including the Australian Physical Activity and Sedentary Behavior Guidelines [23], the guidelines on pregnancy and exercise published by Physical Activity Australia [12], and Dietary Guidelines for Australian Adults [24]. General physical activity advice framed around these guidelines was provided at baseline—at least 30 min of moderate physical activity most days of the week, or 150 min per week. Examples given by Physical Activity Australia include brisk walking, dancing, cleaning windows or sweeping, or pushing a stroller, equating to a level of physical activity of *fairly* or *very* active as measured by the pedometer. Patients were also provided with information regarding step count categorization: a step count <5000 steps/day is classified as sedentary, 5000 to 7499 steps per day as low activity, 7500 to 10,000 steps per day as fairly active, and >10,000 steps as active [13]. This guidance was based on advice from the state tertiary obstetric referral hospital regarding a target of 10,000 steps/day [25] and evidence in obstetric populations that a step count exceeding 10,000 steps/day results in a reduction in GWG [9].

### Data Collection

Baseline demographic data collected directly from patients included age, parity, country of birth, educational background, and household income (previously shown to influence activity levels in pregnancy) [26]. Weight and height were directly measured in the antenatal clinic, and baseline BMI calculated. Activity data collected included daily step counts and daily active minutes (*fairly active* plus *very active* minutes). Absolute GWG (baseline to 36-37 weeks of gestation) was calculated from directly measured weights in the antenatal clinic.

### Outcomes

The primary aim of this feasibility trial was to refine and test the trial protocol for a follow-on large, multicenter trial. Specific feasibility outcomes were recruitment feasibility, engagement and recruitment rate, maintenance of blinding of the control group to pedometer step count (concealment of pedometer display with tamperproof tape), participant compliance with wearing pedometers (days with missing pedometer data) and syncing data regularly, participant retention to study conclusion, and data integrity and completeness of uploaded step counts to investigators. Further secondary aims to guide a definitive multicenter trial were to examine efficacy in increasing step count to a target of 10,000 steps daily in pregnant women with obesity via feedback from the pedometer, evaluate the added benefit of investigator feedback compared with participant self-monitoring alone on the reduction in excessive GWG of participants, and assess the magnitude of any effect to further inform sample size calculation for a definitive trial.

### Statistical Analysis

Data were summarized using mean (SD), median (IQR), or number (%) as appropriate. The baseline variables (age, BMI, and gestation day at recruitment) and outcome variables (steps and activity level) were first examined for the linearity of association. Step count and minutes active were analyzed using a linear mixed model with random intercepts (minutes active) and multiple linear regression using generalized estimating equations (step count), as the maximum likelihood estimation did not converge in the linear mixed model approach for step count. Variables were centered around a gestation of 100 days, BMI of 35 kg/m^2^, and age of 30 years. The linear mixed-model approach allowed controlling for activity level at baseline, as gestational age at enrollment varied between participants. BMI, age, parity at recruitment, and gestational period (classified into months of gestation) were also controlled for in the statistical model. Complete case analysis was used, with observations containing missing data not included in analyses. Statistical analyses were performed using Stata 14.1 (Stata Corp).

### Sample Size Calculation for Definitive Follow-Up Study

No formal sample size was calculated for this feasibility trial. A sample of 30 women was considered adequate to provide data on the feasibility outcomes listed. To test the trial protocol and feasibility endpoints, we aimed to enroll 30 patients. A follow-up definitive randomized controlled two-arm trial sample size was calculated based on the reduction in excess GWG with exercise interventions in women with overweight and obesity contained within the aforementioned Cochrane review [7]; 62% of control patients vs 52% of exercise intervention patients experienced excessive GWG, with a risk ratio of 0.84 (95% CI 0.73 to 0.95). At a power of 0.90 and an alpha error of .05, 533 patients in each group (1066 patients in total) would be required in a follow-up interventional trial.

## Results

**Figure 1:**
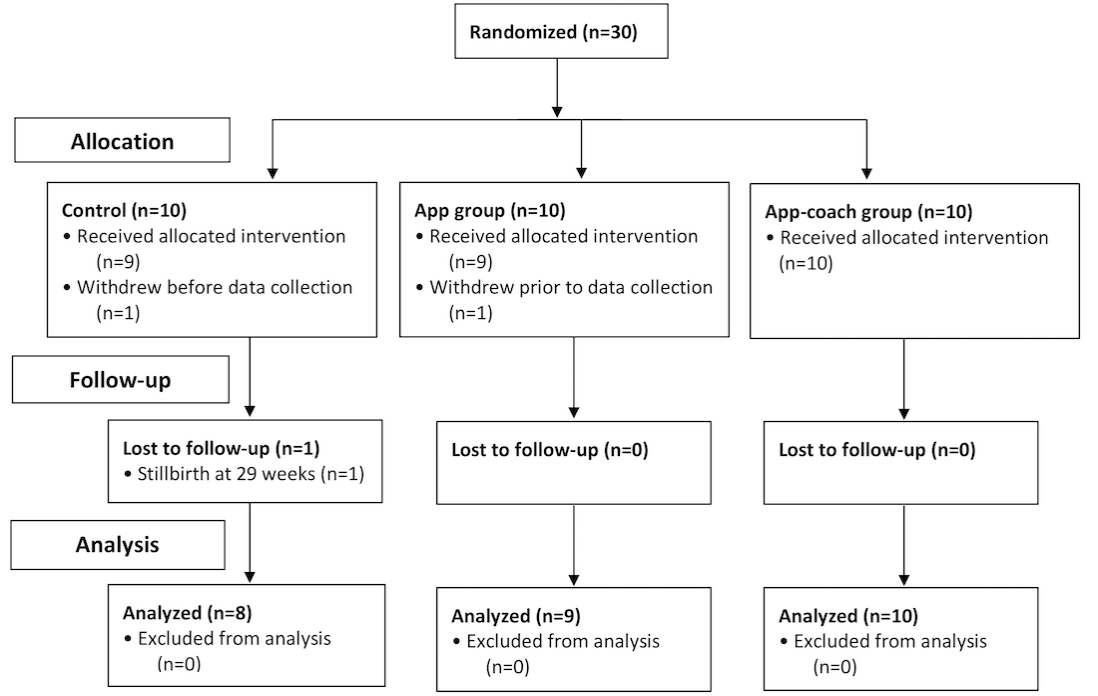
Flowchart of study participants through the trial.

A total of 30 patients (10 per group) were enrolled in the 3 groups (control, app, and app-coach), of whom 2 participants withdrew without activity data recorded (1 from the control group and 1 from the app group) and were subsequently excluded from the analysis. An additional control patient had a stillbirth at 29 weeks of gestation, with no further data collection. Outcome data were thus available for a total of 27 patients (Figure 1). In the app-coach group, 2 patients failed to attend their initial assessments, and no further intervention contact was made. The initial in-person intervention was delivered for the remaining 8 app-coach group patients between gestational week 16 and 20, with 4 patients continuing to completion of all 3 scheduled telephone calls. Group demographics were similar at recruitment, with mean BMI in all groups ≥35 kg/m^2^ (Table 1). There were no differences in country of birth, educational background, and household income between groups.

**Table 1:** Baseline characteristics (total enrolled cohort).

Variable	Control (n=10), mean (SD)	App group (n=10), mean (SD)	App-coach group (n=10), mean (SD)
Age at recruitment (years)	30.2 (5.3)	30.0 (5.0)	28.4 (5.8)
BMI at recruitment (kg/m^2^)	35.9 (4.4)	36.7 (4.4)	37.0 (4.2)
Height (cm)	163.2 (6.0)	166.0 (6.8)	161.2 (8.5)
Weight at recruitment (kg)	95.2 (8.4)	101.5 (16.0)	96.3 (15.6)
Gestation day at recruitment	110 (21.0)	105 (12.0)	112 (19.0)

### Feasibility Outcomes

Recruitment and retention rates were feasible, with all 30 participants recruited within a 10-week period, and a dropout rate of 10% (2 withdrawals and 1 stillbirth). Target population recruitment feasibility was also adequate, with an annual caseload of >1000 pregnant women with obesity seen at Sunshine Hospital. Control group blinding was adequate, with concealment of pedometer display maintained at each check. Patient compliance with wearing pedometers was problematic, with a percentage of days with missing data mean (SD) of 23.4% (20.6%), 39.5% (32.4%), and 21.2% (16.0%) in control, app, and app-coach groups, respectively. Over the study duration, 4 pedometers were lost, requiring replacement. Overall, regular data syncing via automatic mobile phone connection was feasible in app and app-coach group patients, although required troubleshooting in 5 women (1 manually and 4 remotely via telephone).

### Activity Data

**Figure 2:**
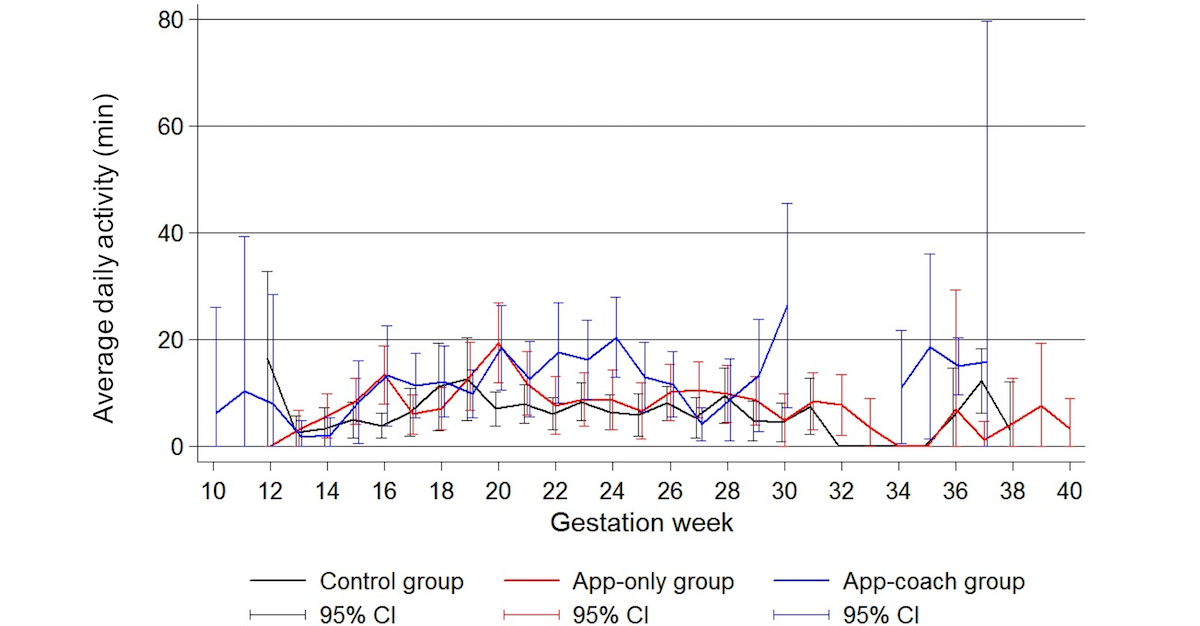
Line plot showing the average (mean and 95% CI) daily activity in minutes by gestation week and treatment group for the 27 patients who completed the study. Note missing activity data in the app-coach group between 30 and 34 weeks.

There was no evidence of a nonlinear association between the baseline and outcome variables. Therefore, the variables were entered into the statistical models without transformation. Results of the linear mixed model investigating activity level are presented in Table 2. The estimated mean baseline daily active minutes for a 30-year-old nulliparous control patient with a BMI of 35 kg/m^2^ and between 61 and 90 gestational days was 14.5 min. Compared with control patients, there was no difference in active minutes for patients in the app or app-coach groups. There were also no significant differences for any group in activity level trends across the gestational period (Figure 2).

A 1-year increase in age was associated with an estimated increase in the daily activity of 0.6 min (95% CI 0.1 to 1.2 min, *P*=.03, and a 1 kg/m^2^ increase in BMI was associated with an estimated reduction in the daily activity of 0.9 min (95% CI 0.3 to 1.5 min, *P*=.005). The estimated effect for parity was each previous live birth being associated with a decrease in the daily activity of 4.5 min (95% CI 0.7 to 8.2 minutes, *P*=.02).

**Table 2:** Fixed-effect estimates from the linear mixed model investigating activity level.

Variable	Coefficient	Standard error	*P* value	95% CI
App group	2.11	3.35	.53	−4.46 to 8.68
App-coach group	1.82	3.20	.57	−4.45 to 8.10
Gestational month	−0.49	0.41	.24	−1.30 to 0.32
BMI	−0.91	0.32	.005	−1.53 to −0.28
Age	0.64	0.29	.03	0.08 to 1.20
Parity	−4.47	1.91	.02	−8.21 to −0.72
Baseline^a^	14.54	3.18	reference	8.31 to 20.76

^a^Baseline represents baseline activity in minutes for an individual in the control group between 61 and 90 gestational days with a BMI of 35 kg/m^2^, 30 years of age, and with no previous births.

### Step Counts

**Figure 3:**
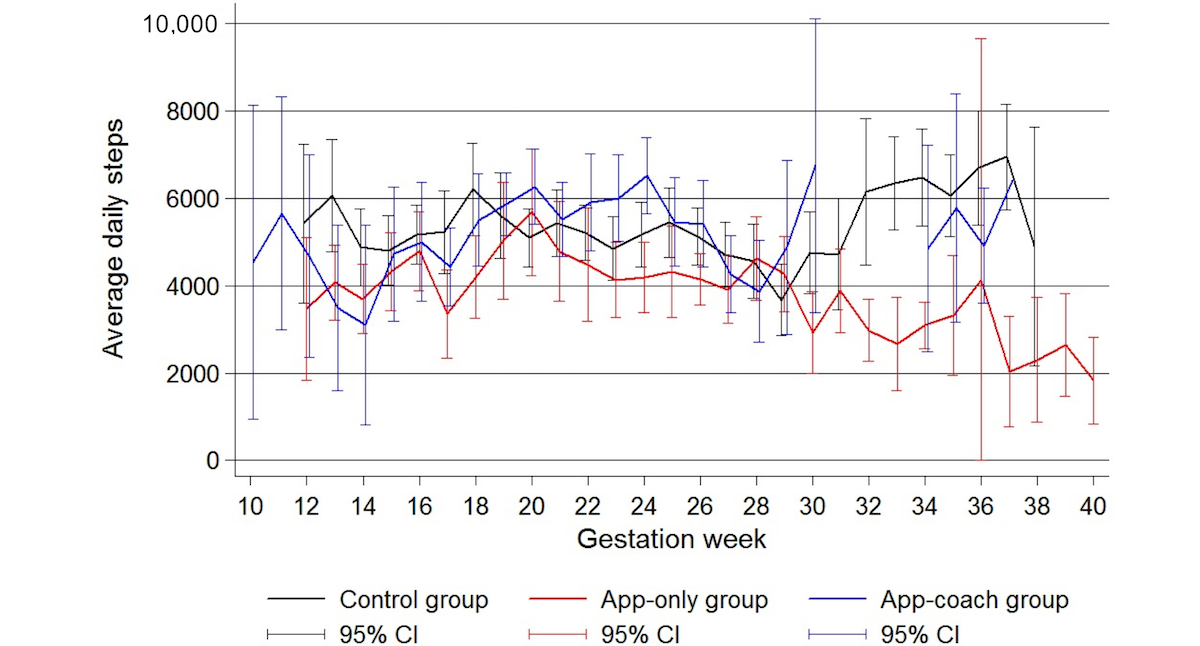
Line plot showing the average (mean) daily steps by gestation week and treatment group for the 27 patients who completed the study with the corresponding 95% CI. Note missing step data in the app-coach group between 30 and 34 weeks.

The estimated mean baseline daily step count for a 30-year-old nulliparous control patient, with a BMI of 35 kg/m^2^ and between 61 and 90 gestational days was 5455 steps (Table 3). Gestation day was the only variable with a statistically significant effect on step count (decrease of 7.80 steps/day for each additional day of pregnancy; 95% CI 2.91 to 12.69, *P*=.002), with no difference in daily step counts between groups. From the 12th to the 29th gestational week, daily step counts did not vary between groups. However, a divergence in daily step trajectories was subsequently observed, with the average daily step count decreasing for the app group compared with participants in either the control or app-coach group, although these differences were nonsignificant (Figure 3). Overall, mean daily step counts in all groups remained in the sedentary (5000 steps/day) or low activity (5000-7499 steps/day) categories for the entire study duration. A step count of over 10,000 daily steps was recorded on 62 days over the study duration, 15 days by 4 control patients, 9 days by 3 app group patients, and 38 days by 6 app-coach patients.

**Table 3:** Results of the statistical model investigating steps.

Variable	Coefficient	Standard error	*P* value	95% CI
App group	−267.02	610.68	.66	−1463.94 to 929.90
App-coach group	661.45	784.76	.40	−876.65 to 2199.55
Gestational day	−7.80	2.50	.002	−12.69 to −2.91
BMI at recruitment	−90.40	71.65	.21	−230.84 to 50.03
Age at recruitment	131.94	82.29	.11	−29.35 to 293.23
Parity	−276.92	367.83	.45	−997.85 to 444.01
Baseline^a^	5454.89	450.22	reference	4572.48 to 6337.30

^a^Baseline represents baseline step count for an individual in the control group between 61 and 90 gestational days with a BMI of 35 kg/m^2^, 30 years of age, and with no previous births.

### Gestational Weight Gain

Mean (SD) GWG was 13.22 (5.91), 7.91 (4.17), and 13.21 (5.73) kg in control, app group, and app-coach group patients, respectively. When allowing for the increased weight at baseline of app group patients, there was no significant difference between groups in GWG, although accounting for the small sample size and resultant significant uncertainty around this estimate, the direction of effect was toward a reduction in weight gained. The results of the multiple linear regression model are shown in Table 4. An increase in weight at recruitment of 1 kg was associated with a further increase in GWG of 0.89 kg (95% CI 0.72 to 1.06 kg, *P*<.001).

**Table 4:** Results of the statistical model investigating gestational weight gain.

Variable	Coefficient	Standard error	*P* value	95% CI
App group	−5.46	2.84	.07	−11.51 to 0.59
App-coach group	−0.40	2.80	.89	−6.37 to 5.57
Weight at recruitment	0.89	0.08	<.001	0.72 to 1.06
Age at recruitment	0.28	0.26	.30	−0.27 to 0.84
Parity	−3.50	1.64	.050	−7.00 to 0.01
Baseline^a^	111.17	2.49	reference	105.87 to 116.47

^a^Baseline represents weight at delivery for a participant in the control group with a weight at recruitment of 95 kg, age at recruitment of 30 years, and no previous births.

## Discussion

### Principal Findings

This randomized, controlled feasibility trial has demonstrated the feasibility of activity data syncing with a personal smartphone in a cohort of pregnant women with obesity. Challenges were demonstrated, however, in the delivery of the behavioral intervention and feasibility of aggregating data because of patient noncompliance with pedometer wearing and loss of devices. These resulted in high missing data rates. Inactivity was common, with baseline activity rates less than half the recommended 30 min/day. Higher BMI in early pregnancy was associated with lower activity levels, as seen in past studies, and increased GWG.

### Relationship to Prior Literature

We observed a comparable level of inactivity with other antenatal populations using pedometer data. A 2011 Australian study examined activity levels of 30 overweight or pregnant women with obesity between 26 and 28 weeks of gestation, reporting a mean (SD) daily step count of 4680 (2520) steps/day [15]. The same group, in the 2014 *HeLP-her* trial, observed a baseline mean (SD) step count of 5438 (3145) steps/day in 98 women at 12 to 15 weeks of gestation [14]. A 2010 Danish study of 338 pregnant women measured comparatively higher overall mean step counts, although lower in women with obesity: 6482, 7446, and 4626 steps/day versus 7558, 8865, and 6289 steps/day in normal-weight women at gestational week 13, 21, and 37, respectively [16]. We observed mean daily step counts significantly lower than these levels, more in keeping with prior Australian studies. Such a difference is possibly related to the high educational levels reported in the Danish study population with potentially increased activity rates. We also observed stable step counts across the gestational period. The reasons for this are unclear but are likely related to already-sedentary baseline activity levels in early pregnancy in our cohort.

Our study differed from previous pedometer-based activity interventions in the novel methodology of automated data-upload, with much-improved data integrity and completeness (overall mean days with complete data ranging between groups from 60.5% to 78.9% of all study days) compared with previous study methods. A randomized follow-up intervention by the aforementioned Danish group, for example, randomly allocated 425 pregnant women with obesity to increased pedometer-guided physical activity, compared with standard antenatal care [27]. In this study, step counts were self-recorded and reported; thus, only half of the participants reported any step count data and only for one-quarter of the study period. The Australian HeLP-her trial was similarly constrained, with pedometers being periodically worn for a specified period only (3-7 days) and the generated data extrapolated to estimate total physical activity [14]. Although our study provides a much more comprehensive picture of activity throughout pregnancy, we also observed considerable missing data rates because of patients forgetting to wear the pedometer and loss of the device, both likely related to the small pedometer size. There is thus the opportunity for future protocol refinement, such as incorporating modern smartphones directly, which have inbuilt ability to measure activity data. Recent studies have demonstrated the validity of these devices in measuring step counts, which may lead to even greater data completeness given the likely improved compliance with carrying and reduced chance of losing a personal smartphone [28,29].

We observed no difference in step counts, daily active minutes, or weight gain reduction between groups, although this feasibility trial was underpowered to assess this. Evidence suggests a major challenge is improving outcomes in a pregnant cohort already with obesity. A 2015 Cochrane review of dietary and exercise interventions in 11,000 women across 49 randomized controlled trials consistently found benefits for women with normal BMI, but no significant reduction in pregnancy weight gain for overweight or women with obesity [7]. A future definitive trial will have to overcome these challenges, likely through achieving greater engagement with a behavioral change program than was seen in this initial trial. More flexibility in the delivery of the intervention, timing to coincide with regular scheduled antenatal appointments, or batch-delivery in a group setting are all strategies that could be explored in improving engagement in a definitive trial. Our cohort also had more obesity than in the aforementioned HeLP-her trial, with a mean baseline BMI of 35.9, 36.7, and 37.0 kg/m^2^ among groups, versus 30.3 and 30.4 in control and intervention groups in the HeLP-her trial, respectively. This may explain the disparate finding in our study, of an association with baseline BMI and increased GWG, compared with an inverse correlation in the HeLP-her trial. The significantly reduced activity levels found with increasing BMI in our cohort may explain this difference, illustrating further challenges if comparable magnitudes of obesity are observed in a definitive follow-up study population.

### Implications of the Study Findings

The findings of this study imply that wearing a pedometer with the ability to sync data with a personal smartphone is feasible in pregnant women with obesity. These findings also imply that future studies seeking to improve physical activity via wearable devices should focus on ways to improve compliance and engagement with the intervention. This study found no benefit to an additional individualized behavioral intervention from clinicians, although lower than expected engagement makes this conclusion uncertain, and this study was not powered to assess this.

### Strengths and Limitations

Strengths of this study include the novel study design using the combination of cheap and robust wearable devices with mobile phones, which are ubiquitous in a younger participant cohort in contemporary Australian society (the overall population smartphone ownership in 2018 was 89%) [30]. An additional strength of this study was the ability to successfully deliver the intervention to those women who engaged with the process. A major limitation of our study was missing data, potentially making conclusions around activity, step data, and GWG endpoints less precise. Encouragingly, data syncing and upload from patients’ smartphones did not appear to be a factor. Rather, the combination of lack of pedometer wearing and outright loss of the pedometer were major contributors, all likely related to the pedometer’s small size and lack of integration in patients’ daily habits. Refining our trial methodology to step-counts measured directly by the newer generation of smartphones, with inbuilt activity apps, would be beneficial. Although phones may be similarly affected by noncarrying time, it is likely that there would be less overall missing data using these devices. An alternative strategy would be the use of *reminders* that are native to the smartphone operating systems or embedded within the fitness apps to prompt participants to sync data more regularly. Another strategy would be to use such reminders to improve compliance with pedometer wearing, which could be extended to direct researcher-participant contact in the event of identified poor compliance.

A further limitation was our stratification of missing data by censoring at 1000 steps/day, although there is no accepted, validated definition in the mobile health literature for what constitutes a day without appropriate pedometer usage. We note that <1000 steps/day has been used by previous studies to define a nonvalid pedometer day [31]. Other studies have used activity time as a surrogate marker of wearing, defining days with <3 hours of data recorded as missing, and 3 to 8 hours of data as half-days [14]. This approach, however, has the potential to erroneously exclude sedentary periods (during which time activity data are not being recorded, despite the pedometer being worn effectively). We could have enhanced our missing data analysis with pedometer wearing diaries, although we note that compliance with these has been shown to be poor [14]. Complete case analysis was also used, meaning observations that contained missing data were not used. This could potentially have biased results, though we have no reason to believe that missing data rates were not distributed randomly among groups or across the gestational period.

There are also potential inaccuracies inherent in wearable pedometers, although we note a recent systematic review of Fitbit pedometers commented favorably on the measurement of steps in adults with no mobility restrictions, as in our cohort [32]. The same review did caution against inaccuracies in the active minutes measured, with a tendency to underestimating sedentary time. We also did not collect information on diet and calorie intake, which may have influenced overall GWG, although we have no reason to believe that this varied between groups. A final major limitation was the difficulty in delivering the behavioral change intervention, with only 4 of 10 women following through to intervention completion. This resulted in significantly reduced group separation, and limits the generalizability of our findings, with the possibility that true differences because of either intervention were not revealed in this feasibility trial.

### Conclusions

This study suggests that activity data syncing with a personal smartphone is feasible in a cohort of pregnant women with obesity, although our results do not support a follow-up study with this design. A future definitive study seeking to reduce GWG and improve activity in this population must focus on improving compliance with activity data recording and with the behavioral intervention delivered. Greater flexibility in intervention delivery for patients and improvements in activity monitoring through direct use of participant smartphones are strategies to explore before a definitive trial.

## Abbreviations

GWG: gestational weight gain

## References

[1] McIntyre HD, Gibbons KS, Flenady VJ, Callaway LK (2012). Overweight and obesity in Australian mothers: epidemic or endemic?. Med J Aust.

[2] (2016). The Royal Australian and New Zealand College of Obstetricians and Gynaecologists.

[3] Goldstein RF, Abell SK, Ranasinha S, Misso M, Boyle JA, Black MH, Li N, Hu G, Corrado F, Rode L, Kim YJ, Haugen M, Song WO, Kim MH, Bogaerts A, Devlieger R, Chung JH, Teede HJ (2017). Association of gestational weight gain with maternal and infant outcomes: a systematic review and meta-analysis. J Am Med Assoc.

[4] Rasmussen KM, Yaktine AL, Institute of Medicine, National Research Council Committee to Reexamine IOM (2009). Pregnancy weight guidelines. Weight Gain During Pregnancy: Reexamining The Guidelines.

[5] Cedergren MI (2007). Optimal gestational weight gain for body mass index categories. Obstet Gynecol.

[6] Callaway LK, Prins JB, Chang AM, McIntyre HD (2006). The prevalence and impact of overweight and obesity in an Australian obstetric population. Med J Aust.

[7] Muktabhant B, Lawrie TA, Lumbiganon P, Laopaiboon M (2015). Diet or exercise, or both, for preventing excessive weight gain in pregnancy. Cochrane Database Syst Rev.

[8] Ehrlich SF, Sternfeld B, Krefman AE, Hedderson MM, Brown SD, Mevi A, Chasan-Taber L, Quesenberry CP, Ferrara A (2016). Moderate and vigorous intensity exercise during pregnancy and gestational weight gain in women with gestational diabetes. Matern Child Health J.

[9] Jiang H, Qian X, Li M, Lynn H, Fan Y, Jiang H, He F, He G (2012). Can physical activity reduce excessive gestational weight gain? Findings from a Chinese urban pregnant women cohort study. Int J Behav Nutr Phys Act.

[10] (2011). World Health Organisation.

[11] Harrison CL, Brown WJ, Hayman M, Moran LJ, Redman LM (2016). The role of physical activity in preconception, pregnancy and postpartum health. Semin Reprod Med.

[12] (2019). Physical Activity Australia.

[13] Tudor-Locke C, Bassett DR (2004). How many steps/day are enough? Preliminary pedometer indices for public health. Sports Med.

[14] Harrison CL, Lombard CB, Teede HJ (2014). Limiting postpartum weight retention through early antenatal intervention: the HeLP-her randomised controlled trial. Int J Behav Nutr Phys Act.

[15] Harrison CL, Thompson RG, Teede HJ, Lombard CB (2011). Measuring physical activity during pregnancy. Int J Behav Nutr Phys Act.

[16] Renault K, Nørgaard K, Andreasen KR, Secher NJ, Nilas L (2010). Physical activity during pregnancy in obese and normal-weight women as assessed by pedometer. Acta Obstet Gynecol Scand.

[17] Dodd JM, Grivell RM, Crowther CA, Robinson JS (2010). Antenatal interventions for overweight or obese pregnant women: a systematic review of randomised trials. Br J Obstet Gynaecol.

[18] Gal R, May AM, van Overmeeren EJ, Simons M, Monninkhof EM (2018). The effect of physical activity interventions comprising wearables and smartphone applications on physical activity: a systematic review and meta-analysis. Sports Med Open.

[19] Darvall JN, Parker A, Story DA (2016). Feasibility and acceptability of remotely monitored pedometer-guided physical activity. Anaesth Intensive Care.

[20] (2019). FitBit.

[21] Locke EA, Latham GP (2002). Building a practically useful theory of goal setting and task motivation. A 35-year odyssey. Am Psychol.

[22] Ryan RM, Deci EL (2000). Self-determination theory and the facilitation of intrinsic motivation, social development, and well-being. Am Psychol.

[23] (2019). Australian Government Department of Health.

[24] (2019). Eat For Health.

[25] (2019). The Royal Women's Hospital.

[26] Renault K, Nørgaard K, Secher NJ, Andreasen KR, Baldur-Felskov B, Nilas L (2012). Physical activity during pregnancy in normal-weight and obese women: compliance using pedometer assessment. J Obstet Gynaecol.

[27] Renault KM, Nørgaard K, Nilas L, Carlsen EM, Cortes D, Pryds O, Secher NJ (2014). The treatment of obese pregnant women (TOP) study: a randomized controlled trial of the effect of physical activity intervention assessed by pedometer with or without dietary intervention in obese pregnant women. Am J Obstet Gynecol.

[28] Amagasa S, Kamada M, Sasai H, Fukushima N, Kikuchi H, Lee I, Inoue S (2019). How well iPhones measure steps in free-living conditions: cross-sectional validation study. JMIR Mhealth Uhealth.

[29] Höchsmann C, Knaier R, Eymann J, Hintermann J, Infanger D, Schmidt-Trucksäss A (2018). Validity of activity trackers, smartphones, and phone applications to measure steps in various walking conditions. Scand J Med Sci Sports.

[30] (2019). Deloitte.

[31] Houser NE, Donald SW, Kolen AM (2019). The impact of valid pedometer days on average daily steps and wear time in children. J Phys Act Res.

[32] Feehan LM, Geldman J, Sayre EC, Park C, Ezzat AM, Yoo JY, Hamilton CB, Li LC (2018). Accuracy of FitBit devices: systematic review and narrative syntheses of quantitative data. JMIR Mhealth Uhealth.

